# Optical assessment of silicon nanowire arrays fabricated by metal-assisted chemical etching

**DOI:** 10.1186/1556-276X-8-216

**Published:** 2013-05-07

**Authors:** Shinya Kato, Yasuyoshi Kurokawa, Yuya Watanabe, Yasuharu Yamada, Akira Yamada, Yoshimi Ohta, Yusuke Niwa, Masaki Hirota

**Affiliations:** 1 Department of Physical Electronics, Tokyo Institute of Technology, Meguro-ku, Tokyo 152-8552, Japan; 2 PRESTO, Japan Science and Technology Agency (JST), Honcho, Kawaguchi, Saitama 332-0012, Japan; 3 Photovoltaics Research Center (PVREC), Tokyo Institute of Technology, Tokyo 152-8552, Japan; 4 Advanced Materials Laboratory, Nissan Research Center, Kanagawa, Yokosuka 237-8523, Japan

**Keywords:** Silicon nanowire, Optical confinement, Light scattering, Solar cells, 73.25.+i, 77.55.df, 78.67.Uh

## Abstract

Silicon nanowire (SiNW) arrays were prepared on silicon substrates by metal-assisted chemical etching and peeled from the substrates, and their optical properties were measured. The absorption coefficient of the SiNW arrays was higher than that for the bulk silicon over the entire region. The absorption coefficient of a SiNW array composed of 10-μm-long nanowires was much higher than the theoretical absorptance of a 10-μm-thick flat Si wafer, suggesting that SiNW arrays exhibit strong optical confinement. To reveal the reason for this strong optical confinement demonstrated by SiNW arrays, angular distribution functions of their transmittance were experimentally determined. The results suggest that Mie-related scattering plays a significant role in the strong optical confinement of SiNW arrays.

## Background

Silicon nanowire (SiNW) arrays demonstrate considerable promise as an absorber layer for solar cells because of their advantages such as quantum size effect [1] and strong optical confinement [2-6]. Many researchers have investigated the optical properties of SiNW arrays fabricated by several methods such as metal-assisted chemical etching (MAE) [7-9], vapor–liquid-solid method [10], laser ablation [11], thermal evaporation [12], and reactive ion etching [13]. Some researchers have reported the control of diameter and density of SiNW arrays using self-assembled close-packed 2-D arrays of nano/microparticle arrays or nanopatterns, and so on. Recently, SiNW solar cells have been extensively investigated for the utilization of their optical confinement [14-16] properties. Vertically aligned SiNW arrays exhibit low reflection and strong absorption [5] and can be used in antireflection coatings or as the active layer in solar cells [17,18]. The optical properties of such arrays investigated thus far have included the influence of silicon substrates. The optical properties of vertically aligned SiNW arrays have been theoretically evaluated by several researchers [3,4,19]. On the other hand, Bao et al. reported that SiNW arrays with random diameter show significant absorption enhancement [19]. According to this paper, we focused on SiNW arrays fabricated by the MAE method to enhance absorption in SiNW arrays with random diameter. To apply these arrays to large-area solar cells, many researchers have adopted SiNW arrays by MAE method, and SiNW arrays prepared by the MAE method tend to have nanowires with a broad range of diameters and may contain bundles of nanowires that adhere to each other due to the wet etching process [7]. Although the optical properties of SiNW arrays have been reported, their light-scattering properties have been scarcely investigated. It is essential to investigate the light-scattering properties of SiNW arrays in order to understand their high optical confinement. In this study, we have investigated the optical properties of SiNW arrays prepared by MAE. Since the SiNW arrays prepared by this method are deposited on silicon substrates, it is difficult to measure the optical properties of SiNW arrays in isolation from the substrate. To remove the effect of the substrate, the SiNW arrays were peeled from the substrate. We present experimentally determined angular distribution functions (ADFs) [20] of the transmittance of SiNW arrays composed of SiNWs of different lengths. The effects of light scattering were also investigated.

## Methods

The silver nanoparticles were fabricated by electroless silver plating. Si wafers (p-type, (100), 2 to 10 Ω·cm) were immersed in a silver coating solution composed of 0.015 M AgNO_3_ and 4.8 M HF for 1 min to cover the surface with silver nanoparticles. The size of the silver nanoparticles appears in the range of 20 to 60 nm. The silver nanoparticle-coated Si wafers were placed in an etching solution composed of 4.8 M HF and 0.15 M H_2_O_2_ at room temperature. The length of the resulting SiNW arrays was controlled by the etching time. In this time, the etching time was varied from 5 to 10 min. After etching, the wafers were dipped in a HNO_3_ aqueous solution for 10 min to remove all remaining silver nanoparticles. The wafers were then immersed in a 5% HF solution to remove the oxide layer. After preparation of the SiNW arrays, polydimethylsiloxane (PDMS) solution [21] was spin-coated on the arrays at 200 rpm and baked at 150°C. The transmittance of the 2-mm-thick PDMS coating was more than 90% in the range from 400 to 1,100 nm and exhibited a refractive index of about 1.4. The SiNW arrays thus embedded in the PDMS coating were mechanically peeled from the substrate with a razor blade.

**Figure 1:**
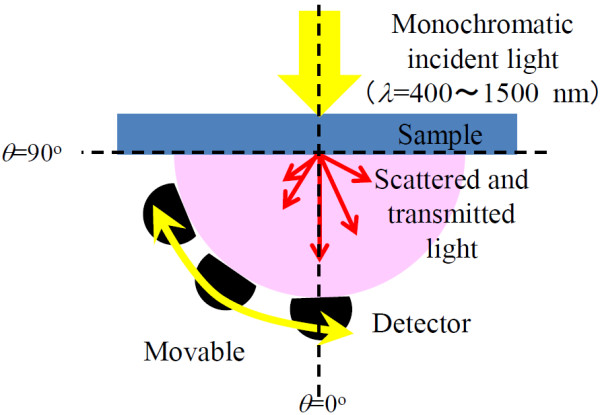
Schematic diagram of an angle-resolved scattering measurement.

The optical properties of the peeled SiNW arrays were measured by an ultraviolet–visible-near-infrared spectrophotometer (Shimadzu Solid Spec-3700, Kyoto, Japan). The spectrophotometer was equipped with a unit for measurement of the ADF as illustrated in Figure 1. The ADF defines the intensity distribution of scattered light as a function of the angle at which the scattered light propagates. The wavelength of the incident light was varied from 400 to 1,500 nm. The detector was moved from 0° to 90° in 5° increments. The structure of the SiNW arrays before and after they were peeled from the substrate was characterized by field emission scanning electron microscopy using a JEOL JSM-7001F instrument (Akishima-shi, Japan). The length of SiNW arrays after peeling off was determined by a scanning electron microscopy (SEM) image.

## Results and discussion

**Figure 2:**
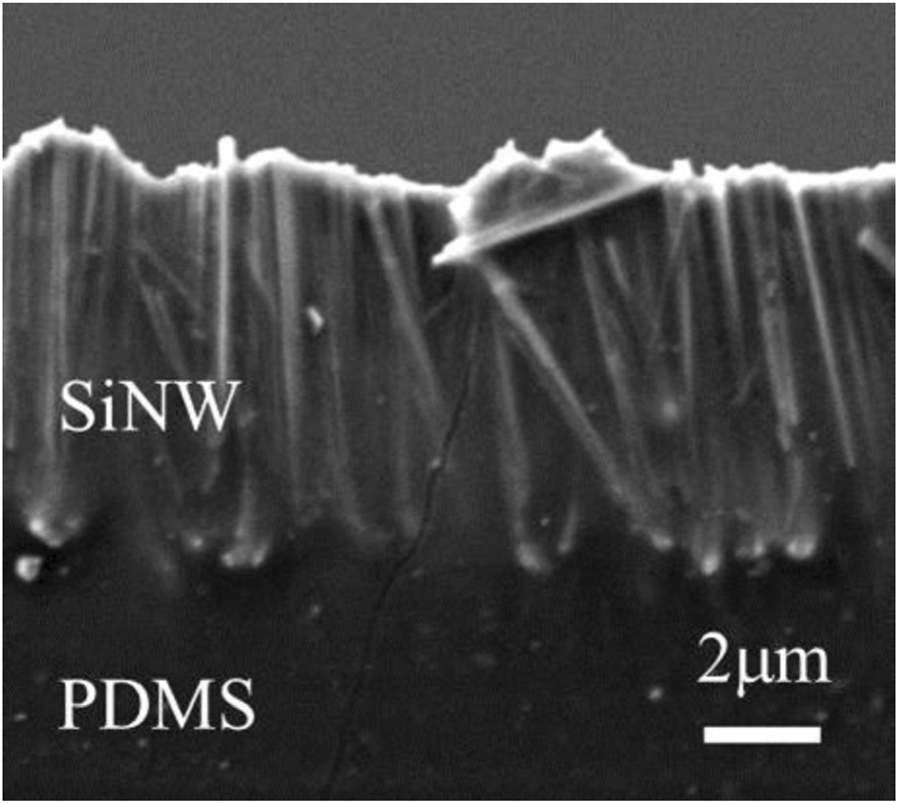
**Cross-sectional SEM image of a SiNW array.** The SiNW array encapsulated in a PDMS matrix has been peeled off from a silicon substrate.

**Figure 3:**
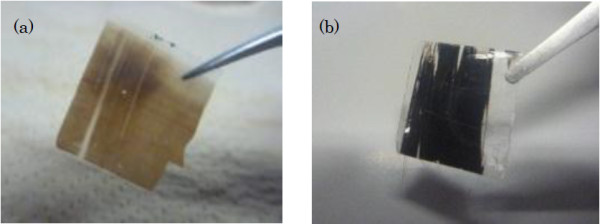
**Photographs of the SiNW array peeled from silicon substrates.** The lengths of SiNWs in the arrays pictured are (**a**) 1 μm and (**b**) 10 μm, respectively.

**Figure 4:**
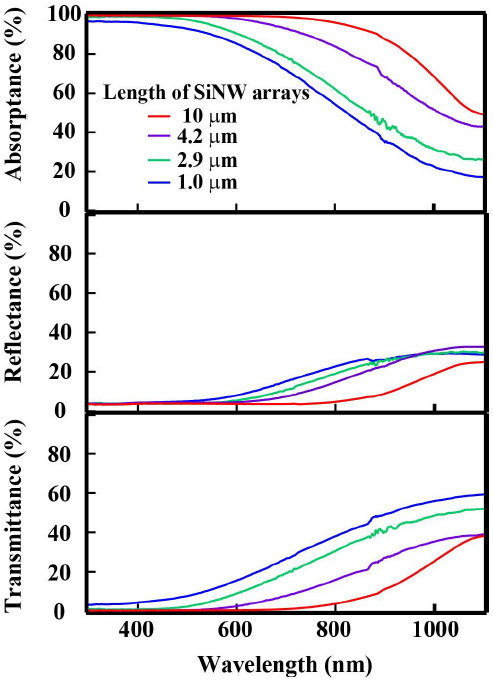
**Absorptance, reflectance, and transmittance spectra of SiNW arrays.** The SiNW lengths of 1.0, 2.9, 4.2, and 10 μm.

Figure 2a shows a cross-sectional SEM image of a SiNW array embedded in a PDMS matrix after being peeled from the substrate. The SEM image indicates that the SiNW/PDMS layer has sufficient mechanical strength to allow the SiNW array to be successfully peeled from the silicon substrate. Moreover, from the SEM images, it was confirmed that the shape of SiNW arrays was maintained, and the diameter of the SiNWs was determined to be 30 to 150 nm. Figure 3 provides photographs of peeled SiNW arrays having SiNW lengths of (a) 1 μm and (b) 10 μm. It can be observed from Figure 3 that the SiNW/PDMS composite composed of 10-μm-long SiNWs appears black, whereas the SiNW/PDMS composite composed of 1-μm-long SiNWs appears brown. This result indicates that the absorption of the SiNW/PDMS composite composed of 1-μm-long SiNWs was low over the visible spectrum. Figure 4 shows the absorptance, reflectance, and transmission of various SiNW arrays having 1.0-, 2.9-, 4.2-, and 10.0-μm-long nanowires along with the theoretical absorption of a 10-μm-thick flat Si wafer calculated using the absorption coefficient of the bulk silicon. To remove the influence of reflectance, the absorptance (*A*) can be represented by:

(1) $$A=1-\frac{T}{\left(1-R\right)},$$

where *T* is the transmittance and *R* is the reflectance. Generally, absorptance is calculated by *A* = 1 − *R* − *T*. However, in this time, the calculated *A* includes the effect of surface reflection. Since the surface reflection was determined by the refractive indexes of air and PDMS, it is not essential to understand the absorption enhancement due to a scattering effect by SiNW arrays. Since we would like to focus on the absorption enhancement due to the scattering in SiNW arrays, we divided *A* by 1 − *R* to assume that the intensity of an incident light right after entering into the SiNW array (to remove the effect of surface reflection) is 1. Although the array with 1-μm-long SiNWs sufficiently absorbed wavelengths below 400 nm, absorption began to decrease for wavelengths greater than 400 nm and was reduced to 50% at 680 nm. The absorption of the array with 1-μm-long SiNWs was calculated as the short circuit current (*I*_sc_) on the assumption that all solar radiation below 1,100 nm was converted to current density and *I*_sc_ is 25.7 mA/cm^2^. It can be observed from Figure 4 that the absorption of SiNW arrays increased with increasing SiNW length. In the case of the SiNW array with the length of 10 μm, it is enough to absorb the light in the whole region and *I*_*sc*_ is 42 mA/cm^2^, which is almost the same value as that of the limiting current density. Therefore, if an array with 10-μm-long SiNWs were to be applied to a solar cell, the solar cell would be expected to exhibit high efficiency.

**Figure 5:**
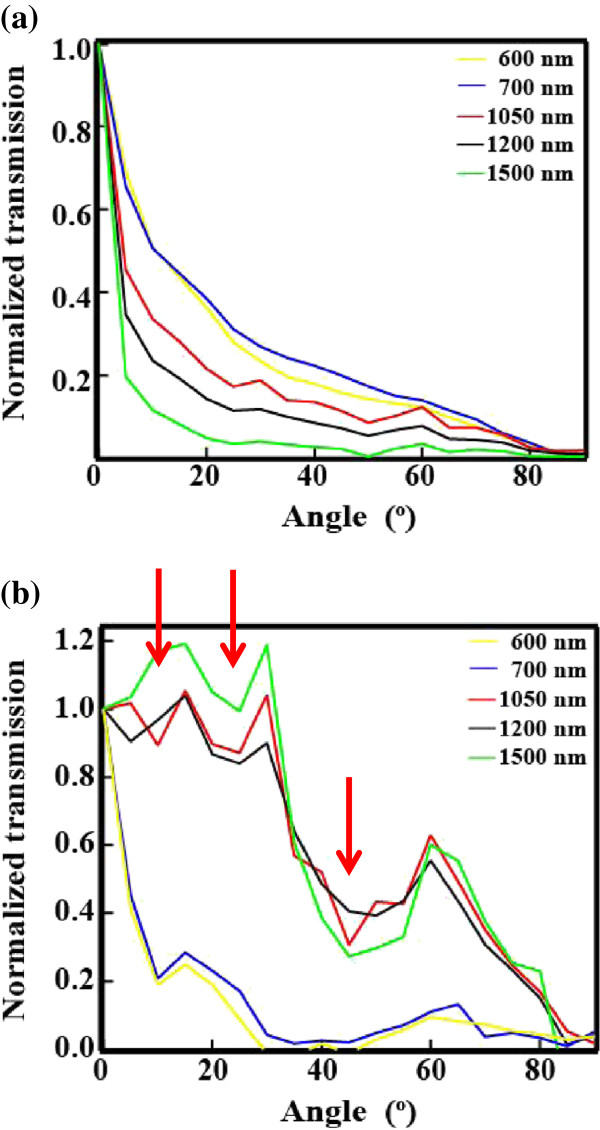
ADF of transmittance of SiNWs with lengths of (a) 1 μm and (b)10 μm.

**Figure 6:**
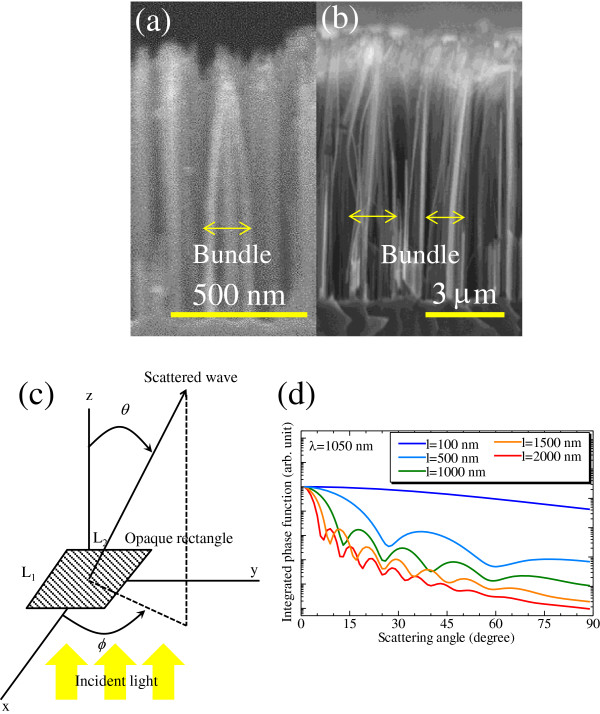
**Cross-sectional SEM images of SiNW arrays attached to silicon substrates.** (**a**) 1-μm- and (**b**) 10-μm-long arrays. (**c**) A diagram of the calculation model of an opaque rectangular obstacle illuminated by a plane wave. (**d**) Integrated phase function at a wavelength of 1,050 nm for various length opaque rectangular obstacles.

To investigate the reason why SiNW arrays demonstrate such strong optical confinement, their scattering properties were evaluated. Figure 5 shows the ADF of transmittance for the SiNW arrays having nanowire lengths of (a) 1 and (b) 10 μm. This result was calculated as the average of s-wave and p-wave incidence, i.e., for unpolarized incidence. In the case of the array with 1-μm-long SiNWs, the transmittance at *θ* = 0° is the strongest for all wavelengths. This trend is similar to that observed for conventionally textured zinc oxide thin films [20]. Figure 5a indicates that the transmittance increased slightly at scattering angles greater than 50° as the wavelength approached the length of the SiNWs. On the other hand, in the case of the array with 10-μm-long SiNWs, for incident light above the wavelength of approximately 1,000 nm, the ADF range demonstrating large transmittance was expanded toward higher scattering angles. Since higher transmittance over larger scattering angles leads to the enhancement of photocurrent, the array with 10-μm-long SiNWs demonstrates a high absorption coefficient for wavelengths above approximately 1,000 nm. Another prominent feature illustrated by Figure 5b is that the ADF exhibits several local minima around 10°, 25°, and 45°. These length-dependent ADF features may be explained by the structure of the SiNW arrays. The long SiNWs, such as the 10-μm-long ones, have a tendency to form bundles after the wet etching process because of the surface tension during drying, as shown in the SEM images in Figure 6a, b for the 1 and 10 μm SiNWs, respectively. From the SEM images, the lateral size of one bundle of SiNWs with the lengths of 1 and 10 μm is about 0.05 to 0.2 and 1 to 3 μm, respectively. Provided that the space between SiNWs is completely filled with the PDMS matrix, the refractive index of the bundle can be determined by the effective medium approximation because the diameter of the SiNWs is sufficiently smaller than the wavelength of the incident light. It is assumed that one bundle of SiNWs is an opaque rectangle, as shown in Figure 6c. According to the diffraction theory, when an opaque rectangle with the sides of *L*_1_ and *L*_2_ scatters light, the amplitude of the scattered wave is given by:

(2) $$S\left(\theta ,\varphi \right)=\frac{\left(1+\cos\theta \right)}{\pi }\frac{x\sin\left(x\sin\theta \sin\varphi \right)}{x\sin\theta \sin\varphi }\frac{\mathrm{x\gamma}\sin\left(\mathrm{x\gamma}\sin\theta \cos\varphi \right)}{\mathrm{x\gamma}\sin\theta \cos\varphi }$$

where *γ* is the ratio of two sides (*L*_1_/*L*_2_) and $x=\frac{2\mathrm{N\pi}}{\lambda }\frac{L_{2}}{2}$[22], and where *N* is the index of refraction. The phase function *p*(*θ*, *φ*) = |*S*(*θ*, *φ*)|^2^/4*x*^2^*γ* is the fraction of the total scattered light that is scattered into a unit solid angle about a given direction (*θ*, *φ*). When *S*(*θ*, *φ*) becomes zero, *p*(*θ*, *φ*) will also be zero, leading to local minima. The angle at each local minimum is represented by

(3) $$\;\theta_{\min}=\sin^{-1}\left(\frac{\mathrm{n\pi}}{\mathrm{x\gamma}}\right)\;\left(n=1,2,\cdots \right)$$

Figure 6d shows the results of the calculation of the integrated phase function $\int_{0}^{2\pi }p\left(\theta ,\varphi \right)\;\mathrm{d\varphi}$ for *λ* = 1,050 nm when the length of the two sides of an opaque rectangle is varied from 100 to 3,000 nm. In this calculation, *L*_1_ = *L*_2_ = *l*, and *l* has a Gaussian distribution with a half bandwidth of 0.1 *l*. The refractive indices were set at the average values of 3.56 and 1.4 using the effective medium approximation. It is apparent from Figure 6d that as the size of an opaque square increases, the number of local scattering angle minima also increases. There is no local minimum at *l* = 100 nm because the size is sufficiently smaller than the wavelength. In the size range above the wavelength, some local minima exist, and the angle was determined by Equation 3. This trend is similar to that of scattering by a sphere, i.e., Mie scattering [23]. The local minima shown in Figure 5b for a wavelength of 1,050 nm are similar to the minima of the integrated phase function given in Figure 6d for *l* = 1,500 nm, which is also in good agreement with the size of the SiNW bundle illustrated in Figure 6b. This suggests that the strong light confinement observed in SiNW arrays is derived from Mie-related scattering, and it is important to adjust the apparent size of SiNWs to the wavelength of the incident light.

## Conclusions

We succeeded in measuring the key optical properties of SiNW arrays that were prepared with metal-assisted chemical etching and separated from the substrates by peeling. The absorptance of a SiNW array composed of 10-μm-long nanowires is much higher than the theoretical absorptance of a 10-μm-thick flat Si wafer. Therefore, SiNW arrays demonstrate a strong optical confinement effect. To investigate the reason why SiNW arrays demonstrate such a strong optical confinement, their scattering properties were observed. For an array with 10-μm-long SiNWs, the range of high transmittance was expanded to high scattering angles for wavelengths above 1,000 nm. Since high-angle scattering leads to the enhancement of photocurrent, the 10-μm-long SiNW array demonstrates strong light confinement for wavelengths above 1,000 nm. This enhancement of light scattering may be due to Mie-related light scattering because the ADF of this array is similar with the scattering patterns calculated by Mie-related theories.

## Competing interests

The authors declare that they have no competing interest.

## Authors' contributions

SK, YK, YW, and YY carried out experiments and calculations. AY supervised the work and finalized the manuscript. YO, YN, and MH gave the final approval of the version of the manuscript to be published. All authors read and approved the final manuscript.
